# Off-label use of rituximab in refractory pediatric rheumatic diseases: a single-center experience

**DOI:** 10.1186/1546-0096-12-S1-P343

**Published:** 2014-09-17

**Authors:** Francisca Aguiar, Joana Abelha-Aleixo, Mariana Rodrigues, Iva Brito

**Affiliations:** 1 Pediatric Rheumatology Unit, São João Hospital, Oporto, Portugal; 2 Rheumatology, São João Hospital, Oporto, Portugal

## Introduction

Rituximab (RTX) is a chimeric monoclonal antibody directed against the CD20 antigen on the surface of B lymphocytes. It binds to CD20 and causes B cells death by different mechanisms. In what concerns rheumatic diseases, it is only approved for the treatment of rheumatoid arthritis. However, there is growing evidence of its utility in other diseases, including in pediatric patients.

## Objectives

To describe the efficacy, tolerability and safety of RTX in patients with juvenile rheumatic diseases attending our Pediatric Rheumatology Unit.

## Methods

Retrospective review of medical records of patients that were treated with RTX between January 2009 and May 2014. The information collected included: age at diagnosis and at the time of initial treatment with RTX, gender, race, baseline rheumatic disease, previous treatments, indication for RTX, regimens used, follow-up time and data for evaluation of efficacy and adverse events.

## Results

We included five patients, four female, four caucasian and one black child. Four of them had juvenile systemic lupus erythematosus (jSLE) and one had extended oligoarticular juvenile idiopathic arthritis (JIA). The median age at diagnosis was 10 years (range 1 - 17) and median evolution time until receiving RTX was 6 years (range 5 months – 15 years). Previous treatments included high-dose prednisolone (N=5), methylprednisolone pulses (N=5), cyclophosphamide pulses (N=3), methotrexate (N=2), hydroxycloroquine (N=3), mycophenolate mofetil (N=3), azathioprine (N=2), leflunomide (N=1) and etanercept (N=1). The indication for receiving RTX was refractory class IV lupus nephritis in 3 patients, jSLE with refractory multisystem involvement in one patient and severe polyarthritis in a patient with JIA who had had anti-TNFα-induced lupus-like nephritis. The regimens used were variable. The median follow-up time after receiving RTX was 1 year (range 4 months – 5 years). In general, the response to treatment was satisfactory: all the patients showed clinical and analytical improvement after 3 – 12 months. In three patients there was deterioration of the disease between 7 and 14 months after initiation of RTX, and two of them received a second cycle with favorable results. It should be noted that during and/or after RTX patients with jSLE continued therapy with mycophenolate mofetil and prednisolone and the patient with JIA continued corticosteroids, but it was possible to gradually reduce the dose of these drugs. In what concerns adverse events, it was recorded only one case of respiratory tract infection, which was resolved with antibiotics without any complications.

## Conclusion

RTX is an anti-CD20 antibody with off-label use in various rheumatic diseases, including in pediatric population. In this case series we found an overall favorable response to RTX, although some patients needed to repeat RTX pulses more than 6 months after the first treatment, which may be related to its mechanism of action. The drug was well tolerated with only one respiratory tract infection reported. Limitations of this study include its retrospective nature, small sample size and short follow-up period. In conclusion, RTX may be a plausible therapeutic choice in rheumatic diseases with more severe and refractory course.

## Disclosure of interest

None declared

